# Happy Marriages

**Published:** 1854-12

**Authors:** 


Happy Marriages.—We clip the following official table from an English paper, giving a view of the connubial bliss in the city of London:

| | |
|---|---|
| Runaway wives . . . . . . | 1,132 |
| Runaway husbands . . . . . . | 2,348 |
| Married persons legally divorced . . | 4,175 |
| Living in open warfare . . . . . | 17,345 |
| Living in private misunderstanding . . | 13,279 |
| Mutually indifferent . . . . . . | 55,340 |
| Regarded as happy . . . . . . | 3,175 |
| Nearly happy . . . . . . . | 127 |
| Perfectly happy . . . . . . | 13 |

N. B. The above census was taken by an old bachelor.—Ed.

